# Cellulase–lactic acid bacteria synergy action regulates silage fermentation of woody plant

**DOI:** 10.1186/s13068-023-02368-2

**Published:** 2023-08-04

**Authors:** Zhumei Du, Seishi Yamasaki, Tetsuji Oya, Yimin Cai

**Affiliations:** 1 College of Animal Science and Technology, Yangzhou University, Yangzhou, 225009 People’s Republic of China; 2 Japan International Research Center for Agricultural Sciences (JIRCAS), Tsukuba, Ibaraki 305-8686 Japan

**Keywords:** Co-occurrence microbial network, Enzyme–bacteria synergy, Silage fermentation, Single-molecule real-time, Woody plant

## Abstract

**Background:**

Feed shortage is an important factor limiting livestock production in the world. To effectively utilize natural woody plant resources, we used wilting and microbial additives to prepare an anaerobic fermentation feed of mulberry, and used PacBio single-molecule real-time (SMRT) sequencing technology to analyse the “enzyme–bacteria synergy” and fermentation mechanism.

**Results:**

The fresh branches and leaves of mulberry have high levels of moisture and nutrients, and also contain a diverse range of epiphytic microorganisms. After ensiling, the microbial diversity decreased markedly, and the dominant bacteria rapidly shifted from Gram-negative *Proteobacteria* to Gram-positive *Firmicutes*. Lactic acid bacteria (LAB) emerged as the dominant microbial population, resulting in increased in the proportion of the carbohydrate metabolism and decreased in the proportion of the amino acid and “global and overview map” (GOM) metabolism categories. The combination of cellulase and LAB exhibited a synergistic effect, through which cellulases such as glycanase, pectinase, and carboxymethyl cellulase decomposed cellulose and hemicellulose into sugars. LAB converted these sugars into lactic acid through the glycolytic pathway, thereby improving the microbial community structure, metabolism and fermentation quality of mulberry silage. The GOM, carbohydrate metabolism, and amino acid metabolism were the main microbial metabolic categories during ensiling. The presence of LAB had an important effect on the microbial community and metabolic pathways during silage fermentation. A “co-occurrence microbial network” formed with LAB, effectively inhibiting the growth of harmful microorganisms, and dominating the anaerobic fermentation process.

**Conclusions:**

In summary, PacBio SMRT was used to accurately analyse the microbial network information and regulatory mechanism of anaerobic fermentation, which provided a scientific basis for the study of woody silage fermentation theory. This study reveals for the first time the main principle of the enzyme–bacteria synergy in a woody silage fermentation system, which provides technical support for the development and utilization of woody feed resources, and achieves sustainable livestock production.

## Highlights

1. Mulberry is potential feed resource as a natural woody plant.
2. Wilting method inhibits anaerobic fermentation of clostridia during ensiling.
3. Bacteria–enzyme synergy action regulates silage fermentation of woody plant.
4. Microbial community and metabolism regulate the woody silage fermentation.
5. Microbial sequencing provides accurate microbial network and fermentation information.

## Background

With the continued development of the global economy and increasing size of the human population, the demand for animal products such as meat, eggs and milk continues to grow [1]. Regarding animal husbandry production, the output of grass and forage crops does not meet the animal feed requirements, such that livestock production in many countries, including Japan, is dependent on imported feed [2]. Therefore, there is an urgent need to develop new natural biomass resources, such as nutrient-rich woody plants, to overcome the feed shortage caused by the rapid development of animal husbandry [3].

Mulberry (*Morus alba* L.) is a deciduous tree or shrub of the genus *Morus* of *Moraceae*, which grows up to 15 m high. Mulberry is native to central and northern China, but is also cultivated in India, Vietnam, Korea, Japan, Mongolia, many central Asian countries, Russia, and some European countries [4]. The protein content of mulberry is higher than that of grass. It is rich in biologically active substances, amino acids, vitamins, trace elements and other nutrients, and can be used as livestock feed [5, 6]. In addition, mulberry can adapt to various climates and soil types, is resistant to drought, waterlogging and barrenness, and has a fast growth rate, high production capacity and low cultivation cost [7].

Mulberry is usually harvested in the rainy season under conditions of high temperature and humidity, and the moisture content of the fresh branches and leaves can be as high as 80%. Traditional production methods (e.g. hay) may increase the lignin content and lead to a loss of leaves in woody plants [8]. Therefore, the hay preparation method is not suitable for woody plants. The silage fermentation method is considered ideal for preparation and storage, and can solve the above problems [9]. Fermented feed is produced under anaerobic conditions. The epiphytic lactic acid bacteria (LAB) transfer the water-soluble carbohydrate (WSC) in the material into lactic acid, reduce the pH, inhibit the growth of spoilage microorganisms, and allow for effective long-term storage [10]. The moisture content, microbial community, WSC and lactic acid buffering capacity (LBC) of plant materials are important factors in terms of anaerobic fermentation [11–13]. Generally, woody plants have a high moisture content and LBC, but a low WSC and epiphytic LAB, which makes it difficult to achieve high fermentation quality through natural anaerobic fermentation preparation methods [14]. The wilting method is widely used in forage fermentation preparation. The forage moisture is adjusted by wilting, the activity of microorganisms in water is reduced, and the proliferation of microorganisms harmful to silage fermentation, such as *Clostridium*, is inhibited [15]. In addition, microbial additives such as cellulase and LAB inoculants play an important role in improving the quality of fermented feed and are widely used in the production of fermented feed [16]. Cellulolytic enzymes can degrade the cellulose and hemicellulose components in fermented feed into monosaccharides, and LAB can use these sugars to generate lactic acid, reduce the pH of fermented feed, and achieve effective long-term storage.

Silage fermentation is a dynamic process of microbial community succession and metabolite changes [17]. It is difficult to accurately analyse the relationship between the microbiome and fermentation in complex anaerobic fermentation ecosystems using traditional microbial plate culture methods. Next-generation microbial high-throughput sequencing technology has been widely used for studying microbial community structure, but is limited to the microbial genus level [18]. The third generation of PacBio single-molecule real-time (SMRT) sequencing measures longer sequence reads at the species level than the previous generations, thus increasing the sensitivity and accuracy of microbial classification [19].

To effectively utilize woody plant resources to prepare high-quality fermented feed, fresh (FMB) and wilted (WMB) mulberry fermented feed were prepared using cellulolytic enzymes and LAB as additives. PacBio SMRT sequencing was applied to explore the microbial community, co-occurrence network, “enzyme–bacteria synergy” and anaerobic fermentation mechanism of mulberry.

## Methods

### Woody plant and silage fermentation

Mulberry (Memo Sang Nonglin No. 10) was cultivated in an experimental field (140°07′ east longitude, 36°08′ north latitude; Tsukuba, Japan) of the Institute of Agrobiological Sciences, National Agriculture and Food Research Organization (NARO). The experimental field was located in a subtropical maritime monsoon climate zone. In 2020, the field site had a minimum temperature of − 4 °C, maximum temperature of 36 °C, annual average temperature of 13 °C and annual rainfall of 1336 mm. Mulberry cultivated in the experimental field for animal feed can be harvested 2–3 times a year. The mulberry used in this experiment was harvested with a woody harvester during the juvenile stage from the first cutting on June 12, 2020, and the shoots were all about 1.5 m tall. The harvested materials were divided into two parts; the first part was used directly for making fermented feed, and the second part for wilting fermented feed.

The wilted material was directly exposed to the sun in the field for 4 h, and turned over twice to achieve a moisture content of approximately 60%. The FMB and WMB materials were cut into 1–2 cm pieces. After fully mixing, 500 g samples were taken for three replicates. The samples were placed in an ice box and immediately transported to the laboratory for analysis. The remaining materials were used for the preparation of FMB and WMB fermented feed. The commercial LAB inoculant Chikusou-1 (CH) and *Acremonium cellulase* (AC) enzyme were used as microbial additives. The treatments were as follows: control, CH, AC. and CH + AC (CA). The CH inoculant was freeze-dried powder produced by Snow Brand Seed Co., Ltd (Sapporo, Japan), while the CH strain was *Lactiplantibacillus plantarum*, which is a homofermentative LAB. In accordance with the manufacturer’s guidelines, 5 mg/kg of inoculant power was added on a fresh matter (FM) basis, after which the number of LAB was equivalent to 1.0 × 10^5^ colony-forming unit per gramme (cfu/g).

The AC was produced by *Acremonium cellulolyticus*, and consisted of glucanase, pectinase and carboxymethyl-cellulase (total cellulase activity of 7350 U/g; amount added: 0.01% of the FM). The CH and AC were dissolved in distilled water in proportions of 2% and 1%, respectively, and 1 ml/kg of the FM was sprayed onto the material with an electronic sprayer. The control treatment was sprayed with the same amount of distilled water. Both FMB and WMB fermented feed were prepared using a small-scale fermentation system (260 cm × 380 cm; Hiryu KN type; Asahi kasei, Tokyo, Japan). A 200 g sample of the material was packed into a polyethylene plastic bag, which was sealed with a vacuum sealer. There were five replications for each treatment. The bags were stored at room temperature (22–30 °C). After 60 days of ensiling, the bags were opened and the microbial community, fermentation quality and chemical composition were analysed.

### Analysis of chemical composition and LBC

The FMB and WMB materials and their fermented feed, were dried in an oven at 65 °C for at least 48 h until they reached a constant mass. The dried samples were ground in a high-speed vibrating mill (model T1-200; CMT Co., Ltd, Tokyo, Japan). The dry matter (DM; method 930.15), ash (method 923.03), crude protein (CP; method 990.03) and ether extract (EE; method 920.39) of samples were measured according to the analysis methods of the Association of Official Analytical Chemists [20]. The residual moisture of samples was removed by drying in an oven at 105 °C for at least 2 h, and the chemical composition was calculated relative to the DM content. Ash was produced from the dried powder sample (1 g) by burning at 550 °C for 2 h in a muffle furnace (NMF-215B; Masuda Co., Ltd, Osaka, Japan). The organic matter (OM) content corresponded to the weight lost after ashing. The CP was measured using a Kjeldahl nitrogen/protein analyser (model 1026; Foss Technol. Co., Ltd, Hillerod, Sweden). The CP was converted using nitrogen (conversion factor of 6.25). Neutral detergent fibre (NDF), acid detergent fibre (ADF), and acid detergent lignin (ADL) were measured as described by Van Soest et al. [21]. The NDF and ADF were measured exclusive of residual ash. Heat-stable amylase and sodium sulphite were used for the NDF analytical procedure. The ADL was analysed via the solubilization of cellulose with sulphuric acid. The WSC was analysed by high-performance liquid chromatography (HPLC, LC-2000 plus; JASCO Corporation, Tokyo, Japan), using the method described by Cai [22]. The LBC was measured by the titration method described by Muck et al. [23]. The sample pH was reduced to 4.0 using HCl, and then titrated with NaOH from pH 4.0 to 6.0 (mmol/kg DM).

### Protein decomposition and mineral content analysis

The non-protein nitrogen (NPN) and true protein (TP) contents were determined using methods described previously [24]. The macro-minerals, including calcium, phosphorous, magnesium and potassium, were determined using a wet-ashing method and atomic absorption spectrophotometry (LAMBDA 1050; PerkinElmer, Shelton, CT, USA), as described by Adeloju et al. [25].

### Analysis of silage fermentation

The products of the silage fermentation were analysed using the analysis method for fermented feed described by Cai [22]. The fermented feed sample (10 g) was homogenized in 90 mL of sterilized distilled water and placed in a refrigerator at 4 °C for 24 h. After evenly mixing, the extract was filtered through a quantitative ashless filter paper (5A, 110 mm; Advantec Co., Ltd., Tokyo, Japan). The pH value was determined by a glass electrode pH meter (D-71; Horiba Co., Ltd., Kyoto, Japan).

The ammonia nitrogen (NH_3_-N) content of the fermented feed was analysed using the Kjeltec auto distillation system (2200; Foss Tecator, Höganäs, Sweden), as described by Cai [22]. The fermented feed filtrate was shaken through a cation exchange resin (Amberlite, IR 120B H AG; Organo Corporation, Tokyo, Japan) and centrifuged at 6500×*g* for 5 min at 4 °C. The supernatants were passed through a 0.45 µm filter (DISMIC 13HP; Toyo Roshi Kaisha, Ltd, Tokyo, Japan), and the filtrates were injected into an HPLC system (LC-2000 plus; JASCO Corporation, Tokyo, Japan) to determine the organic acid content in accordance with the feed analysis method described by Cai [22].

### Microbial population analysis

The plate count method was used to analyse the microbial population of the fermented feed, as described by Cai et al. [10]. A 10 g sample was blended with 90 mL of sterilized saline solution (0.85% NaCl) and homogenized for 5 min using a laboratory blender (Stomacher 400; Seward, Worthing, UK). The resulting suspension was serially diluted from 10^–1^ to 10^–8^ using a saline solution, and a 0.05 mL aliquot from each diluted suspension was spread on an agar plate.

The LAB were cultured on de Man, Rogosa, and Sharpe agar medium (MRS; Difco Laboratories, Detroit, MI, USA) for lactobacilli in a rectangular jar (5.0 L, Mitsubishi Gas Chemical Company Inc., Tokyo, Japan) under anaerobic conditions. The LAB colonies grown on MRS agar were Gram-positive, catalase-negative bacteria that mainly produce lactic acid from glucose.

Aerobic bacteria and coliform bacteria were cultured on nutrient agar medium (Nissui-Seiyaku Co., Ltd, Tokyo, Japan) and blue light agar (Nissui-Seiyaku Co., Ltd.) under aerobic conditions. Coliform bacteria could be distinguished from other bacteria by the blue colour of their colonies. Yeasts and moulds were cultured on potato dextrose agar medium (Nissui-Seiyaku Co., Ltd.) with 10% sterilized tartaric acid solution (pH 3.5). All plates were incubated at 30 °C for 2–4 days, and the microbial colonies were reported as viable numbers of microorganisms in cfu/g of FM.

### Bacterial community analysis

#### DNA extraction and SMRT sequencing

To prepare samples for SMRT sequencing, 10 g samples were blended with 90 mL of 0.85% (w/v of NaCl) sterilized saline solution and shaken on a mechanical shaker (FS-003; Tokyo Garasu Kikai, Tokyo, Japan) at 250 rpm in a 4 °C refrigerator for 45 min. The filtrate was filtered with pre-autoclaved four-layer gauze and centrifuged at 10,000 rpm for 10 min to achieve microbial precipitation. DNA from the microbial precipitate were extracted by a DNA kit (D5625-01; Omega, Norcross, GA, USA) [8]. The DNA samples were stored in a − 80 °C freezer for subsequent analysis. The full-length 16S rDNA were amplified by polymerase chain reaction (PCR) using 27F and 1492R primers for SMRT sequencing, and the PCR amplicons were purified, quantified and pooled in equal amounts, as described by Du et al. [26]. The amplified DNA was purified using a SMRTbell Express Template Prep Kit 2.0 (PacBio, Menlo Park, CA, USA) to prepare SMRTbell libraries. The libraries were sequenced on a single PacBio Sequel II 8M cell using the Sequel II sequencing Kit 2.0.

#### Sequencing analysis

The RS_Readsofinsert.1 protocol in SMRT Portal software (version 2.7) (PacBio) was used to process the raw data. The Quantitative Insights Into Microbial Ecology (QIIME) package (version 1.7) was used to remove the low-quality sequences. The extracted high-quality sequences were aligned with 100% sequence identity to obtain representative sequences, using Python nearest alignment space termination (PyNAST) and Clustering and Classification Inference with U-Statistics (UCLUST) [27]. Unique sequences were classified into operational taxonomic unit (OTU) based on a 98.6% threshold identity using the UCLUST algorithm. The Chimera Slayer tool was used to remove potential chimeric sequences from the representative set of OTUs [28]. The different OTUs were classified using the Ribosomal Database Project II database classifier, and the taxonomic information for each OTU representative sequence was annotated based on Bergey's taxonomy at the genus, family, order, class, and phylum levels, and classified according to an 80% minimum bootstrap threshold [29]. The OTUs that occurred only once or twice were discarded.

### Statistical analysis

The abundance-based coverage estimator (ACE), Chao1, Shannon and Simpson indices were calculated by QIIME software to assess alpha diversity [30]. Following OTU clustering, to analyse both shared and unique information for FMB and WMB, as well as their fermented samples. Venn diagrams were produced using open-source tools for R software (R Development Core Team, Vienna, Austria). An R-based tool was used to perform a hierarchical cluster and heat map analysis. A correlation network was also derived using a Python-based tool. The linear discriminant analysis effect size (LEfSe) with the relative abundance data was obtained to determine the significance using Python [31]. To compare functional profiles among groups, the metabolic potential of the microbial community and composition of functional genes were assessed by assigning 16S rRNA marker gene sequences to functional annotations of sequenced metagenomic sequences based on the Kyoto Encyclopedia of Genes and Genomes (KEGG), using Phylogenetic Investigation of Communities by Reconstruction of Unobserved States (PICRUSt), as described by Langille et al. [32].

Analysis of variance (ANOVA) of general linear model was used to examine the differences among samples, and was performed with SPSS software (version 19.0; SPSS Inc., Chicago, IL, USA). *P* < 0.05 was taken to indicate statistical significance.

Data concerning pH, LBC, the microbial population, chemical composition, protein decomposition and macro-mineral content of fresh forage were subjected to one-way ANOVA. Tukey’s honestly significant difference test was used to compare means.

Data concerning the microbial population, fermentation quality and chemical composition of the fermented feed at 60 days were subjected to two-way ANOVA with a fully randomized design, including silage fermentation (*S*) and additive (*A*) (2 × 4) as the main variables. Least significant difference tests were performed using SAS software (SAS Institute, Cary, NC, USA) with the following statistical model:

$$Y_{ijk} = \, \mu \, + \, \alpha_{i} + \, \beta_{j} + \, \alpha \beta_{ij} + \, \varepsilon_{ijk} ,$$

where *Y*_*ijk*_ is the observed value, *μ* is the overall mean, *α*_*i*_ is the silage fermentation effect (*i* = FMB and WMB), *β*_*j*_ is the additive effect (*j* = control, CH, AC and CA), *αβ*_*ij*_ is the silage fermentation × additive interaction effect, and *ε*_*ijk*_ is the error. Mean values were compared using Tukey's test [33].

## Results

The pH, LBC, microbial population, chemical composition, protein decomposition and macro-mineral content in FMB and WMB before ensiling are shown in Table 1. The LBC and pH of FMB and WMB were > 638 mEq/kg of DM and 6.4, respectively. The microbial counts in both materials were 10^4^ cfu/g of FM for LAB, 10^5^ for aerobic bacteria, 10^4^ for coliform bacteria, 10^3^–10^4^ for yeast, and 10^2^ for clostridia and mould. The DM content of FMB was < 21%. After wilting, the DM content of WMB increased by 18%. The CP and WSC contents of FMB and WMB were > 22.1% and > 4.6%, respectively. The OM, EE, NDF, ADF, ADL, and macro-mineral contents (including calcium, phosphorous, magnesium and potassium) in FMB and WMB materials were similar, and did not change greatly during the wilting process.

**Table 1:** The pH, LBC, microbial population, chemical composition, protein decomposition, and macro-mineral content in FMB and WMB before ensiling

Item	Treatment		SEM	*P* value
	FMB	WMB		
pH	6.42b	6.93a	0.01	**
LBC (mEq/kg of DM)	638.59b	671.12a	7.17	*
*Microbial population (cfu/g of FM)*				
Lactic acid bacteria	4.5 × 10^4^	6.1 × 10^3^	–	–
Aerobic bacteria	6.3 × 10^5^	8.3 × 10^5^	–	–
Coliform bacteria	2.8 × 10^4^	2.3 × 10^4^	–	–
Clostridia	5.0 × 10^2^	3.5 × 10^2^	–	–
Yeast	6.3 × 10^3^	1.2 × 10^4^	–	–
Mould	4.3 × 10^2^	2.5 × 10^2^	–	–
*Chemical composition*				
DM (%)	24.59b	48.82a	1.37	**
OM (% of DM)	91.52	90.82	0.18	*
CP (% of DM)	22.21	22.15	0.12	NS
EE (% of DM)	2.82	2.84	0.06	NS
NDF (% of DM)	30.17	32.26	2.53	NS
ADF (% of DM)	24.54	26.75	1.91	NS
ADL (% of DM)	2.26	2.59	0.58	NS
WSC (% of DM)	4.64	4.67	0.05	NS
*Protein decomposition (% of DM)*				
NPN	4.08	3.73	0.08	NS
TP	17.92	17.04	0.72	NS
*Macro-mineral content (g/kg of DM)*				
Calcium	1.67a	1.30b	0.03	**
Phosphorous	0.24	0.24	0.01	NS
Magnesium	0.37a	0.28b	0.02	*
Potassium	2.86	2.85	0.08	NS

FMB, fresh mulberry; WMB, wilted mulberry; LBC, lactic acid buffering capacity; DM, dry matter; cfu, colony-forming unit; FM, fresh matter; OM, organic matter; CP, crude protein; EE, ether extract; NDF, neutral detergent fibre; ADF, acid detergent fibre; ADL, acid detergent lignin; WSC, water-soluble carbohydrate; NPN, non-protein nitrogen; TP, true protein; SEM, standard error of mean; NS, not significant

*P < 0.05; **P < 0.01

^a^^−^^b^Means with different superscript in the same row followed are statistically different (*P* < 0.05)

The microbial population, fermentation quality and chemical composition of FMB and WMB silages are shown in Table 2. After ensiling, LAB became the dominant bacteria in all silages. The LAB counts in both control silages were 10^5^ cfu/g of FM, which increased to 10^6^ and 10^7^ in the CH-, AC- and CA-treated silages of FMB and WMB, respectively. The aerobic bacteria counts in FMB silages were 10^5^ for the control, 10^4^ for CH and AC, and 10^3^ for CA, while in WMB silages, they were 10^3^ for the control and 10^2^ for all additive treatments. The counts of coliform bacteria in the control and AC-treated FMB silages were 10^3^, while they were below the detection level (< 10^2^) in the CH- and CA-treated FMB silages, and in all WMB silages. The clostridia counts were 10^3^–10^4^ in both control silages and 10^3^ in additive-treated FMB silages, and were below the detection level (< 10^2^) in all the additive-treated WMB silages. The yeast counts in the control and AC- and CA-treated FMB silages ranged from 10^3^ to 10^4^, while in the CH-treated FMB silages and all WMB silages, the values were below the detection level. The counts for mould were below the detection level in all silages.

**Table 2:** Microbial population, fermentation quality and chemical composition in FMB and WMB fermented feed

Item	FMB				WMB				SEM	*P* value		
	Control	CH	AC	CA	Control	CH	AC	CA		S	A	S × A
*Microbial population (cfu/g of FM)*												
Lactic acid bacteria	6.0 × 10^5^	6.0 × 10^7^	4.0 × 10^7^	6.3 × 10^7^	1.8 × 10^5^	6.2 × 10^7^	5.9 × 10^7^	6.3 × 10^7^	–	–	–	–
Aerobic bacteria	5.0 × 10^2^	1.0 × 10^4^	1.0 × 10^4^	5.0 × 10^2^	1.7 × 10^4^	5.0 × 10^2^	5.5 × 10^3^	4.0 × 10^3^	–	–	–	–
Coliform bacteria	ND	ND	ND	ND	1.4 × 10^5^	ND	1.5 × 10^3^	ND	–	–	–	–
Clostridia	5.4 × 10^4^	ND	ND	ND	1.7 × 10^3^	ND	ND	ND	–	–	–	–
Yeast	ND	ND	ND	ND	5.0 × 10^4^	ND	7.0 × 10^3^	4.7 × 10^4^	–	–	–	–
Mould	ND	ND	ND	ND	ND	ND	ND	ND	–	–	–	–
*Fermentation quality*												
pH	5.79a	4.16b	3.96b	3.86b	5.85a	4.06b	4.18b	3.94b	0.09	NS	***	NS
Lactic acid (% of FM)	0.28c	0.82b	0.90ab	1.02a	0.20c	0.86ab	0.79b	0.98a	0.04	NS	***	NS
Acetic acid (% of FM)	0.75a	0.52b	0.48b	0.54b	0.43a	0.30bc	0.27b	0.32c	0.02	***	***	*
Propionic acid (% of FM)	0.10	ND	ND	ND	ND	ND	ND	ND	NS	NS	NS	NS
Butyric acid (% of FM)	0.26	ND	ND	ND	0.05	ND	ND	ND	NS	NS	NS	NS
NH_3_-N (g/kg of FM)	0.44	0.23	0.12	0.19	0.46	0.20	0.26	0.18	0.03	NS	***	*
*Chemical composition*												
DM (%)	23.80	25.56	23.27	25.25	40.93a	39.93ab	32.92c	36.32bc	1.27	***	**	*
OM (% of DM)	88.66bc	90.24a	88.15c	89.52ab	89.67a	89.79a	88.66b	89.31ab	0.25	NS	***	*
CP (% of DM)	21.84	22.10	21.99	21.85	21.85	21.89	21.43	21.36	0.41	NS	NS	NS
EE (% of DM)	2.64	2.78	2.72	2.80	2.87	2.79	2.62	2.79	0.26	NS	NS	NS
NDF (% of DM)	30.05a	29.21b	27.71c	28.67c	31.84a	29.98b	27.97c	28.12c	0.78	NS	***	NS
ADF (% of DM)	24.45b	24.26b	23.58a	23.81b	26.49a	26.15a	24.29b	25.91b	0.55	NS	***	NS
ADL (% of DM)	2.12	2.19	2.39	2.40	2.35	2.51	2.90	2.64	0.17	*	NS	NS

FMB, fresh mulberry; WMB, wilted mulberry; CH, *Lactiplantibacillus plantarum*; AC, *Acremonium cellulase*; CA, combination of CH and AC; S, silage fermentation; A, additive; S × A, interaction between silage fermentation and additive; cfu, colony-forming unit; FM, fresh matter; ND, not detected; NH_3_-N, ammonia nitrogen; DM, dry matter; OM, organic matter; CP, crude protein; EE, ether extract; NDF, neutral detergent fibre; ADF, acid detergent fibre; ADL, acid detergent lignin; SEM, standard error of mean; NS, not significant

*P < 0.05; **P < 0.01; ***P < 0.001

^a^^−^^c^Means of inoculation treatments within a row with different superscripts differ (*P* < 0.05)

The CA-treated FMB and WMB silages had the highest lactic acid content, and the CH- and AC-treated both silages exhibited with higher (*P* < 0.001) lactic acid and lower (*P* < 0.01) butyric acid and NH_3_-N contents than control silages. The pH of CH-, AC- and CA-treated FMB and WMB silages were lower (*P* < 0.05) than in their control silages.

The CP content of both silages was higher (*P* < 0.05) than in the control than the additive treatments. The NDF contents of AC and CA-treated FMB and WMB silages were lower (*P* < 0.05) than those of the control and CH-treated silages. The DM, OM, EE, ADF and ADL did not change markedly in the control or additive-treated silages. The *S* significantly (*P* < 0.05) influenced the acetic acid, DM and ADL indices of silages, while *A* significantly (*P* < 0.01) influenced the fermentation quality and chemical composition, except for the CP, EE and ADL indices. The S × A significantly (*P* < 0.05) influenced the acetic acid, NH_3_-N, DM and OM indices.

**Fig. 1:**
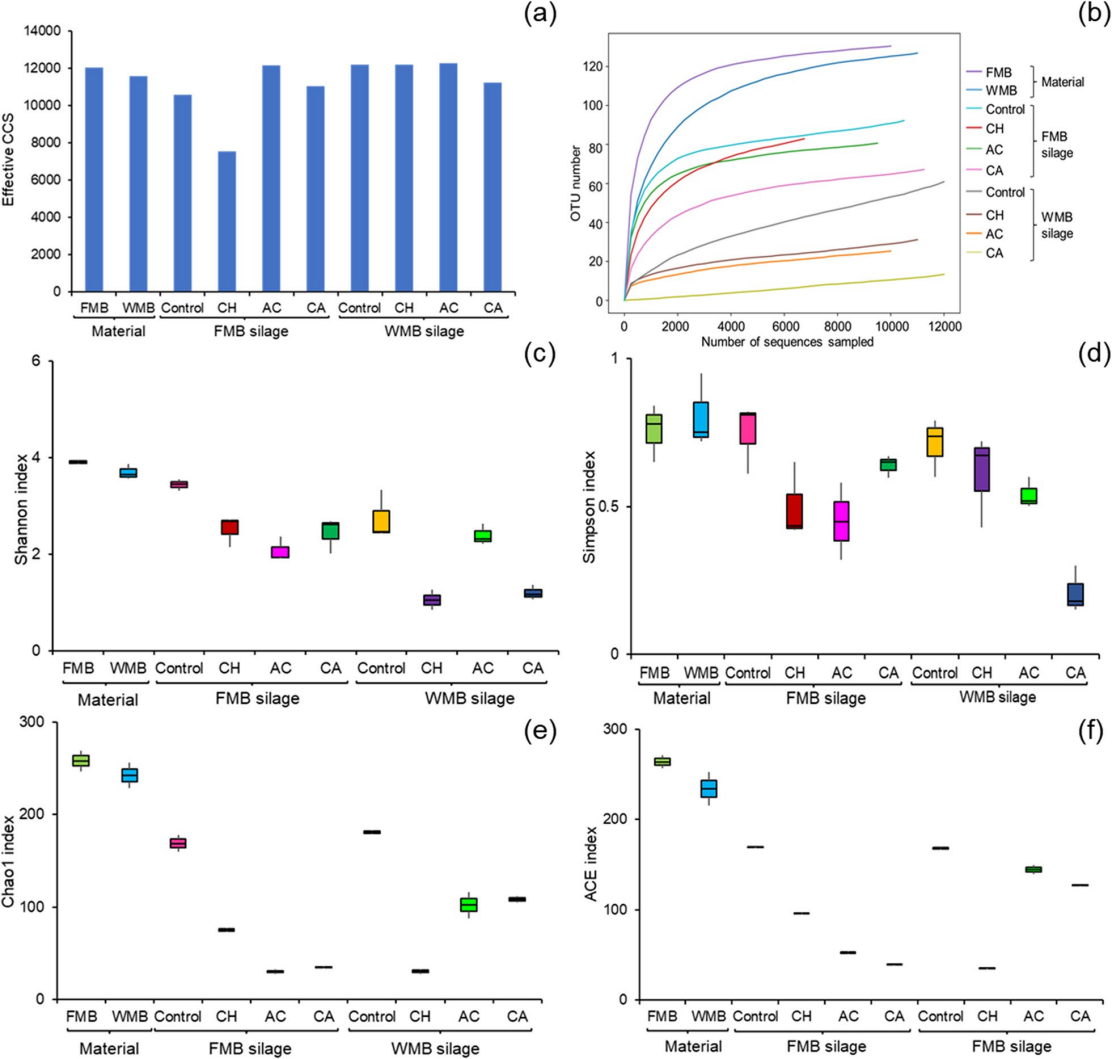
General information of sequence and alpha diversity analysis of material and fermented feed. FMB, fresh mulberry; WMB, wilted mulberry; CH, *Lactiplantibacillus plantarum*; AC, *Acremonium cellulase*; CA, combination of CH and AC; CCS, circular consensus sequencing; OTU, operational taxonomic unit; ACE, abundance-based coverage estimator

The general sequence information and alpha diversity of materials and silages are shown in Fig. 1. The number of optimized circular consensus sequencing (CCS) reads in all treatments ranged from 7547 to 12,272, with an average of 11,259 (Fig. 1a). In the rarefaction analysis for multiple samples, there were approximately 6,500 sequences per sample (Fig. 1b), with a plateau in the number of OTUs detected when a minimum of approximately 250 sequences were rarefied. The number of OTUs in other silages ranged from 20 to 125, with a minimum of approximately 4,000 sequences per sample. Prior to ensiling, the alpha diversity indices (including the ACE, Chao1, Simpson and Shannon indices) were higher in the FMB and WMB materials (Fig. 1c, d, e, f) than in the silages. After ensiling, the alpha diversity indices of the silages decreased, with the microbial additive-treated silages exhibiting less microbial diversity than the control silages.

**Fig. 2:**
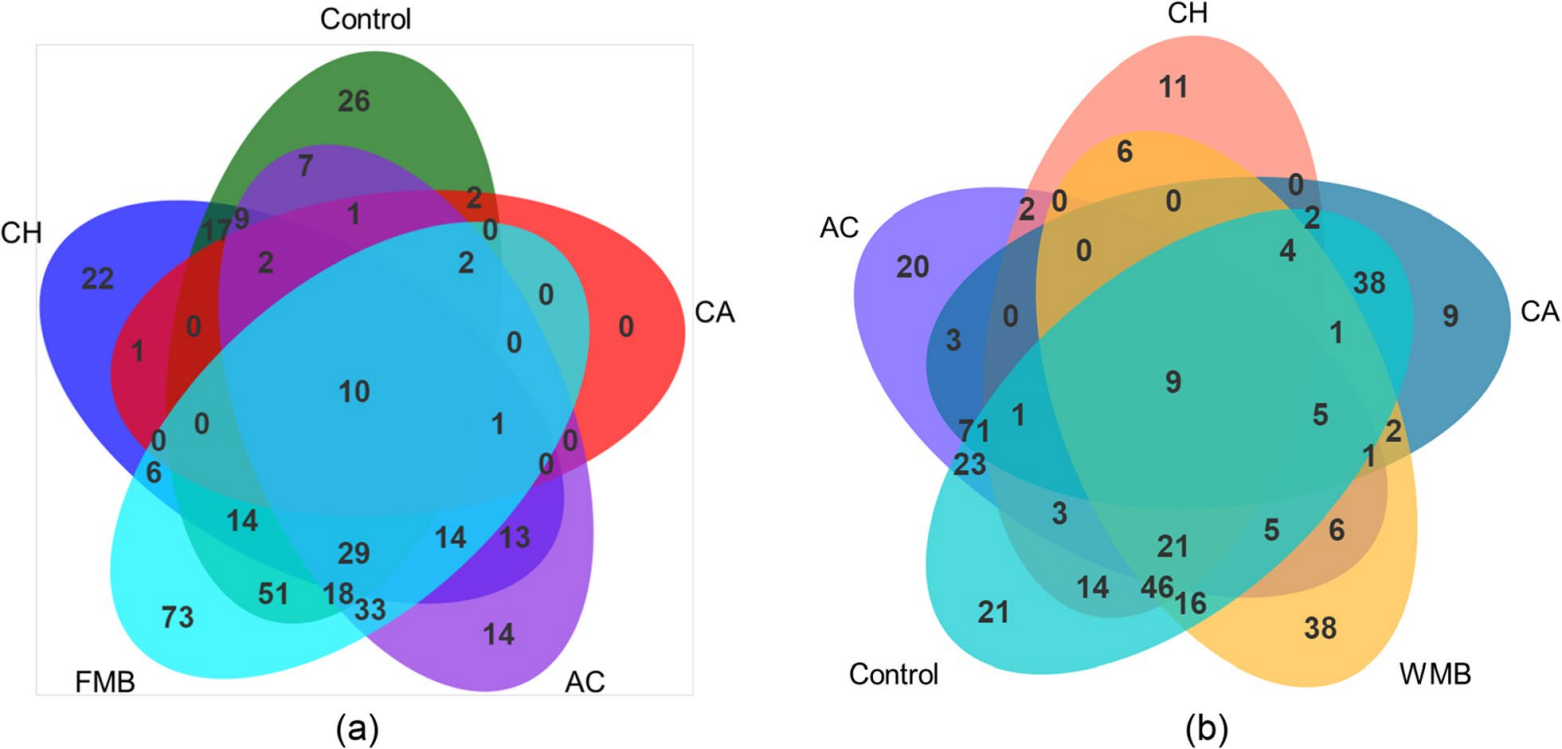
Venn diagram of OTU numbers at 97% sequence identity in FMB (**a**) and WMB (**b**) before and after ensiling. OTU, operational taxonomic unit; FMB, fresh mulberry; WMB, wilted mulberry; CH, *Lactiplantibacillus plantarum*; AC, *Acremonium cellulase*; CA, combination of CH and AC

Venn diagrams of the OTUs at 97% sequence identity in FMB (a) and WMB (b) before and after ensiling are shown in Fig. 2. The OTUs showed trends similar to those observed for the Simpson and Shannon indices. The number of unique bacterial microbiome OTUs identified in FMB and WMB was higher before (Fig. 2a) than after (Fig. 2b) ensiling. The numbers of unique OTUs in FMB and WMB silages were reduced to 47 and 17, respectively, after ensiling. The numbers of core bacterial microbiome OTUs in these two silages were 10 and 9, respectively. Compared to the control, the additive-treated silages had fewer unique OTUs. The numbers of unique OTUs in CH-, AC- and CA-treated FMB and WMB silages were reduced by 4 and 1, respectively, compared to each control.

**Fig. 3:**
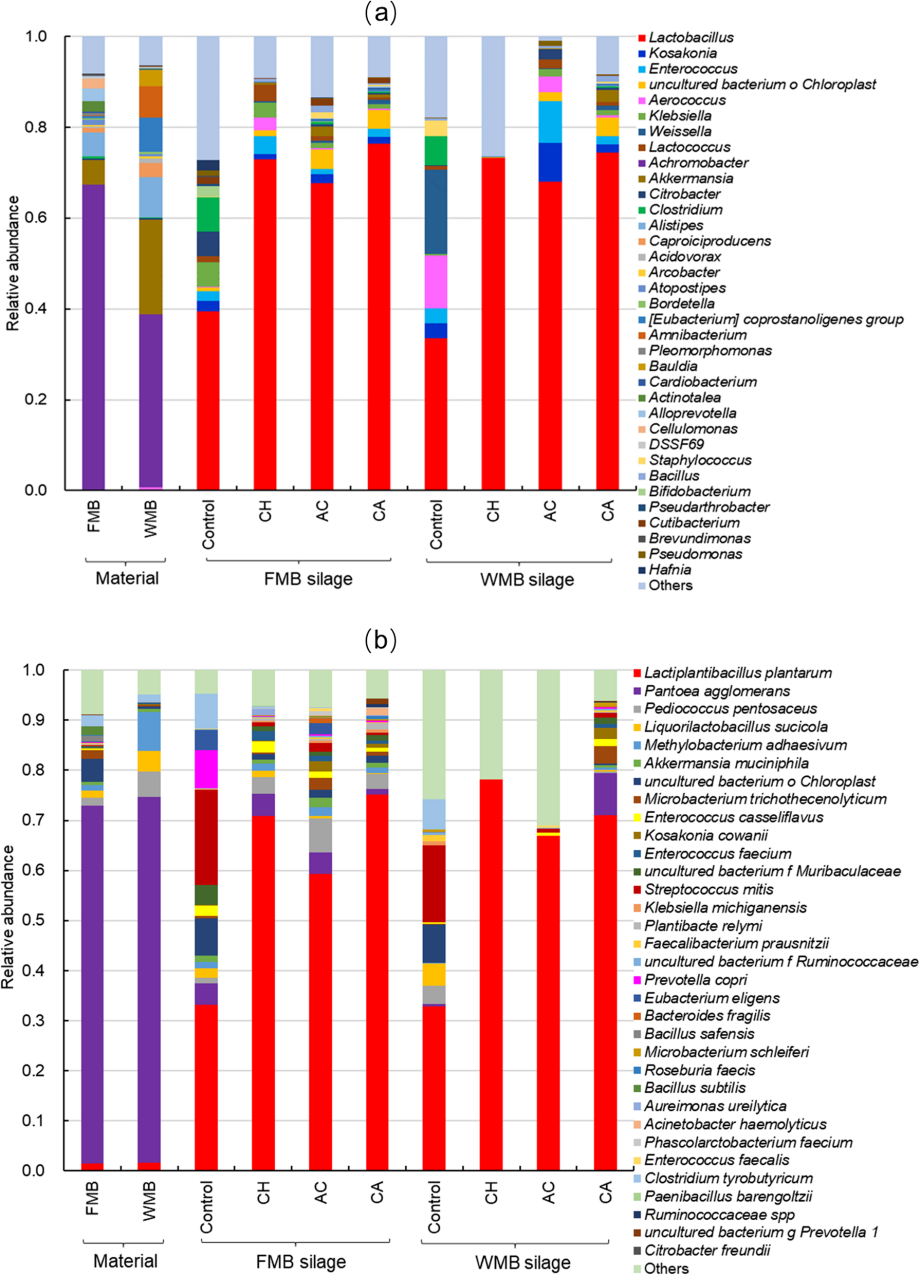
Relative abundance of the top 35 genus (**a**) and species (**b**) levels identified in the bacterial community of the FMB and WMB fermented feed. FMB, fresh mulberry; WMB, wilted mulberry; CH, *Lactiplantibacillus plantarum*; AC, *Acremonium cellulase*; CA, combination of CH and AC. The data for all microbiological samples are the average of three replicates. ^a−c^Means with different superscripts differ (*P* < 0.05). **a** Material; **b** control silage; **c** other additive silage

The relative abundance of the top 35 bacteria in the FMB and WMB silages, determined at the genus and species level before and after ensiling, are shown in Fig. 3. As shown in Fig. 3a, the dominant bacterial genera in FMB and WMB materials were *Achromobacter*, *Akkermansia*, *Alistipes* and *Caproiciproducens*. After ensiling, *Lactobacillus* was the predominant genus in the FMB and WMB silages. The relative abundance of *Lactobacillus* in the additive-treated FMB and WMB silages was higher than in each control silages. A small amount of *Clostridium* was present in both control silages. Compared with the FMB silages, the WMB silages had a higher proportion of *Lactobacillus*. The relative abundance of *Lactobacillus* was higher in the additive-treated FMB and WMB silages than in control silages. *Clostridium* were present in small proportions in the FMB and WMB control silages.

In Fig. 3b, it can be seen that *Pantoea agglomerans* was the dominant species in the FMB material, while both *P. agglomerans* and *Methylobacterium adhaesivum* were dominant in the WMB material. *L. plantarum* was almost undetectable (< 0.02%) in FMB and WMB materials. After ensiling, the most abundant microbial species in all the FMB and WMB silages was *L. plantarum*. The relative abundances of *L. plantarum* were lower in FMB and WMB materials, while higher in both additive-treated silages than those in the control silages. *Clostridium tyrobutyricum* were detected in both control silages, but not in the other silages.

**Fig. 4:**
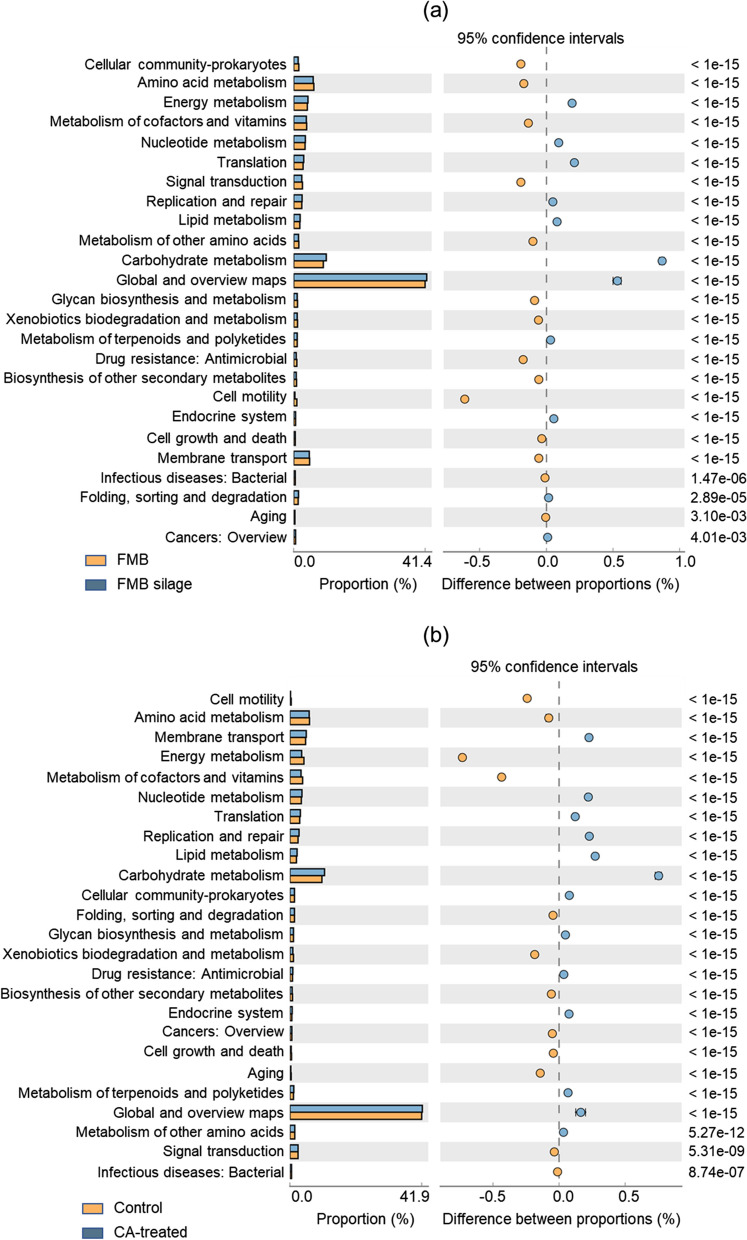
The impacted KEGG metabolism pathway on the second level in mulberry material and fermented feed (**a**), and mulberry fermented feed with or without additives (**b**). KEGG, Kyoto Encyclopedia of Genes and Genomes. FMB, fresh mulberry; WMB, wilted mulberry; CA, combination of *Lactiplantibacillus plantarum* and *Acremonium cellulase*

The significant KEGG metabolic pathways in the FMB material and control silage are shown in Fig. 4a, while the pathways in the control and CA-treated silages are shown in Fig. 4b. Based on the KEGG gene and metabolic pathway analyses, 26 metabolic categories were distinguished after identifying orthologous sequences in the metagenome. The predominant KEGG metabolic categories in FMB material and silages were “global and overview map” (GOM), carbohydrate metabolism and amino acid metabolism. The GOM and carbohydrate metabolism categories were more dominant in FMB silages than FMB material (*P* < 0.001); the opposite trend was seen for the amino acid metabolism pathway (*P* < 0.001). Compared to the FMB control silage, the GOM and carbohydrate metabolism categories in the CA-treated silage were more dominant (*P* < 0.001), while the amino acid metabolism pathway was less dominant (*P* < 0.001).

**Fig. 5:**
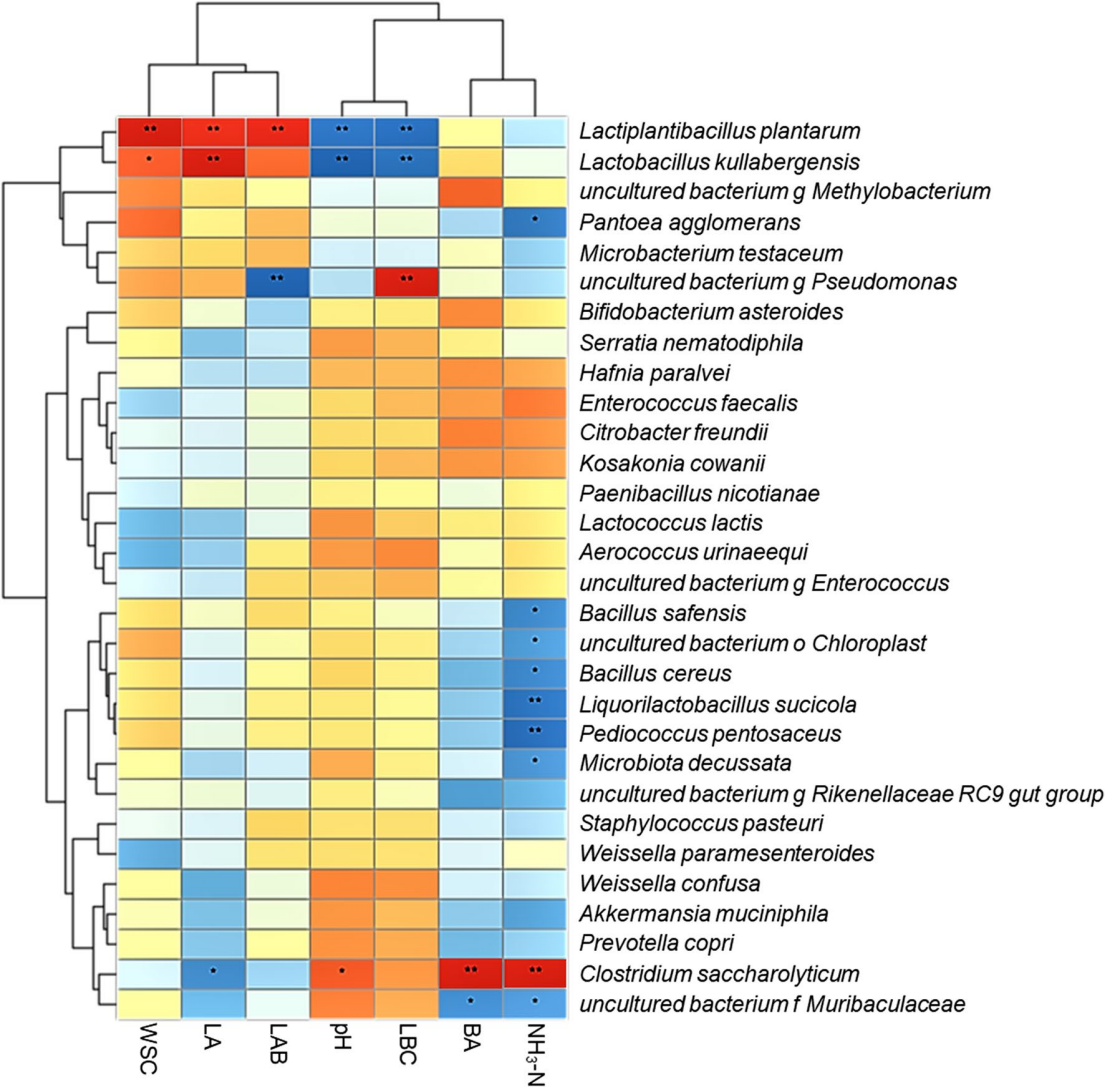
Correlation analysis between bacterial community and terminal fermentation products at the species level. WSC, water-soluble carbohydrate; LA, lactic acid; LAB, lactic acid bacteria; LBC, lactic acid buffering capacity; BA, butyric acid; NH_3_-N, ammonia nitrogen; *P < 0.05; **P < 0.01

The results of correlation analysis between bacterial community and terminal fermentation products at the species level are shown in Fig. 5. There were strong positive correlations of WSC with *L. plantarum* and *Lactobacillus kullabergensis*. Lactic acid was positively correlated with *L. plantarum* and *L. kullabergensis*, but negatively correlated with *Clostridium saccharolyticum*. LAB had a positive correlation with *L. plantarum* and a negative correlation with *Pseudomonas* spp. The pH and LBC were positively correlated with *C. saccharolyticum* and *Pseudomonas* spp., respectively. Both pH and LBC were negatively correlated with *L. plantarum* and *L. kullabergensis*. Butyric acid and NH_3_-N were both positively correlated with *C. saccharolyticum* and negatively correlated with *Muribaculaceae* spp.

**Fig. 6:**
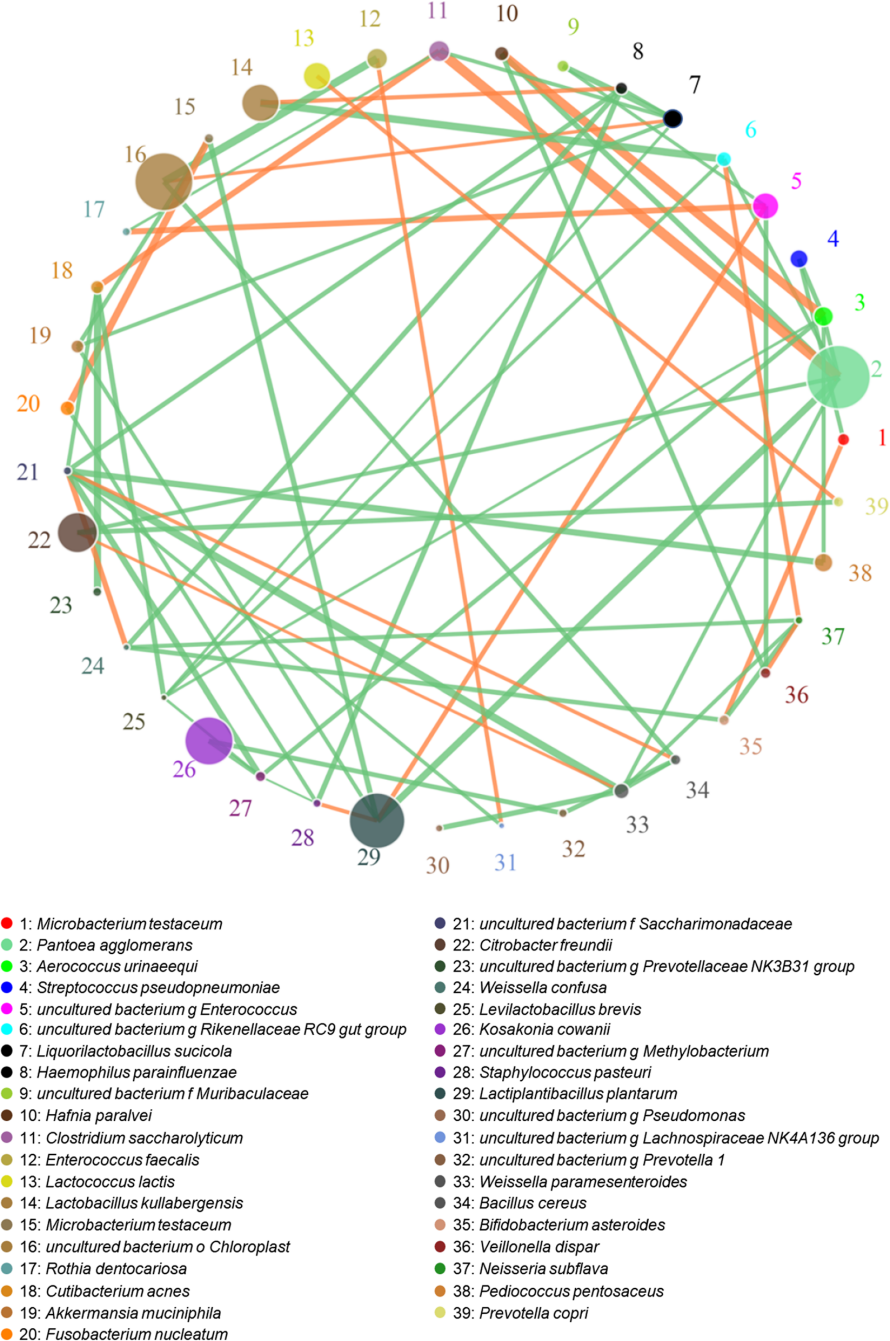
Correlation network analyses among all the microorganisms at species level

The microbial co-occurrence network of FMB silages at the species level is shown in Fig. 6. *L. plantarum* was positively correlated with *Enterococcus* spp., and negatively correlated with *P. agglomerans*, *Microbacterium testaceum* and *Akkermansia muciniphila*. *C. saccharolyticum* was positively correlated with *P. agglomerans* and *Cutibacterium acnes*, and negatively correlated with *Liquorilactobacillus sucicola* and *Rothia dentocariosa*.

## Discussion

### Feed composition and anaerobic fermentation characteristics of mulberry

Many countries, including Japan and China, have low feed self-sufficiency rates, such that livestock production mainly relies on imported feed. To solve the problem of feed shortages and improve the capacity for producing animal products, the use of natural woody feed resources has been proposed. As a potential feed resource, mulberry has a wide distribution, and high adaptability, biomass yield and nutritional value. Moreover, it is palatable to animals, and has therefore attracted extensive attention from animal husbandry researchers and farmers. In-depth study of the chemical composition, fermentation feed preparation methods and storage characteristics of mulberry is important to ensure its effective utilization in animal production. Ruminants generally prefer feeds that are low in fibre and high in CP and amino acids, because these feeds have high nutritional and energy content, and are rich in minerals and vitamins [34]. In vivo and in vitro experiments of mulberry leaf digestion by cattle showed that the digestibility can exceed 80%. In addition, the digestibility of a leaf and branch mixture was similar to that of most tropical forages for ruminants [35]. The chemical composition of mulberry varies by cultivar, tree age, and growing conditions [36]. The CP content in the fresh branches and leaves of mulberry used in this study was > 22% on a DM basis, which the TP proportion was about 80% (Table 1). This is much higher than that of tropical grasses and crop straws, confirming that mulberry has high nutritional value. The ADF content of mulberry leaf is markedly lower than that of tropical grasses [37], and mulberry leaf contains more calcium, phosphorus, magnesium, and potassium, among other minerals. Therefore, mulberry contains large amounts of digestible feed components, and can be used as a high-protein digestible woody feed resource for livestock.

Anaerobic fermentation is considered the ideal method to effectively utilize woody plant resources and cope with feed shortages [26]. The LBC, WSC, moisture and LAB contents of forage materials affect the fermentation quality of feed. For optimal anaerobic fermentation conditions, a 60–70% moisture content, > 6% WSC content (on a DM basis) and > 10^5^ cfu/g LAB content (on an FM basis) are required. If the moisture content is too high, butyric acid fermentation will occur, which reduces the quality of the silage fermentation. However, if the moisture content is too low, the materials should not be trampled to remove residual air, as this will lead to the growth of mould and other aerobic bacteria, which in turn cause fermented feed deterioration. A low WSC content and LAB count are not conducive to lactic acid fermentation, which will also affect the quality of fermented feed. In this study, the moisture of FMB was > 79%; after wilting, it dropped to about 61%, which is ideal for making fermented feed. However, the mulberry feed composition did not change significantly after wilting, thus proving that the wilting method is suitable for adjusting the moisture content of woody plants. The LBC also affects silage fermentation. Woody plants are often rich in cations such as calcium, magnesium, and potassium, and therefore have a high LBC [8]. During ensiling, these cations neutralize the organic acids formed by anaerobic fermentation, thereby hindering pH reduction [38]. In our previous study, the LBC of paper mulberry from southern China was > 900 mEq/kg of DM, and that of woody plants (such as leucaena and gliricidia) from southern Africa was 500–580 mEq/kg of DM, when fermented feed was prepared naturally (i.e. low-quality fermentation) [8, 26]. Therefore, we prepared woody plant fermented feed using local feed resources, such as crop straw and bran, to adjust the LBC of these woody plants and improved their fermentation quality [3]. The LBC of the mulberry used in this study was lower than that of paper mulberry, higher than those of leucaena, gliricidia and other *Poaceae* grasses, and similar to that of alfalfa. Moreover, there were no adverse effects on silage fermentation. The mulberry used in this study, which did not have a very strong LBC (< 680 mEq/kg of DM), could be used to improve the fermentation quality of the silage by wilting and adding microbial inoculants, and we did not adjust the LBC by mixing mulberry with other forages. Some studies have mixed mulberry with other forage crops or grasses and have obtained good fermentation quality similar to this study [39, 40]. The chemical composition and microbiological analysis results for mulberry showed that the epiphytic LAB count and WSC content of FMB and WMB were low, and there were more harmful microorganisms such as enterobacter and clostridia. These chemical and microbial factors are all detrimental to anaerobic fermentation, confirming the importance of applying microbial additives during fermented feed preparation using woody plants [7, 8].

During ensiling, LAB utilizes WSC to produce lactic acid, lower the pH, and inhibit the growth of spoilage microorganisms, thereby preserving the nutrients in the ensiled forage over the long term [12]. As shown in Table 2, after ensiling, the LAB count in all fermented feed increased, while the number of harmful microorganisms (such as aerobic and coliform bacteria) decreased. These results suggest that the fermentation of mulberry fermented feed was similar to that of other forages such as Napier grass, corn stover and sugarcane top [16, 26]. LAB and cellulolytic enzymes were responsible for the substantial improvement seen in the fermentation quality of woody fermented feed, via a synergistic bacteria–enzyme effect. This is because the LAB inoculants used in this study were homofermentative LAB species with strong lactic acid fermentation capacity in fermented feed [41]. These inoculant bacteria can produce a large amount of lactic acid during the fermentation process, and inhibit the growth of harmful microorganisms, thereby improving the quality of fermented feed [10]. The cellulase enzyme used in this study was produced by *A. cellulolyticus*, which contains a variety of enzymes that degrade the plant cell wall such as hemicellulase, pectinase, endogenous and exogenous proteases, amylase and oxidoreductase [26]. Cellulase can degrade structural carbohydrates in materials into monosaccharides or disaccharides, thereby reducing the content of plant cellulose components and providing sufficient fermentation substrates for mulberry fermented feed lacking WSC; this compensates for the lack of sugars in woody plants [42]. By reducing the moisture content of the material, wilting can effectively inhibit the growth of harmful bacteria such as *Clostridium* species, and improve the quality of fermented feed [26]. In the present study, the addition of cellulase decreased the NDF content of mulberry fermented feed due to the decomposition of cellulose components of mulberry by cellulolytic enzymes during ensiling. Some of the water-soluble cellulose was decomposed into WSC, and LAB used these compounds to produce lactic acid, exerting a bacterial-enzyme synergistic effect and improving silage fermentation quality. However, the ADF and ADL contents did not change greatly, regardless of whether cellulase was added. This was because these enzymes did not decompose the indigestible fibre and lignin components during the ensiling process [43]. Therefore, the cellulase used in this study do not decompose ADF and ADL effectively.

### The microbial community and species diversity of material and fermented feed

When analysing microbial diversity, it is necessary to verify whether the amount of sequencing data is sufficient to reflect the species diversity in the sample. Dilution and richness curves can be used to test microbial diversity indirectly. The dilution curve is used to randomly select individuals from a sample, count the number of species therein, and construct another curve representing the number of microorganism species according to the number of individuals. The dilution curve can be used to compare the abundance of microbial species among samples with different amounts of sequencing data, and to determine whether the amount of sequencing data in a sample is reasonable. When the curve is flat, the amount of sequencing data is considered reasonable, and a larger amount of data will only generate a small amount of new OTUs. Additional sequencing may generate more OTUs. By constructing a dilution curve, the sequencing depth can be obtained for a given sample. In this experiment, the flat OTU curve and high number of CCS reads indicated that the sequencing data were reliable (Fig. 1). The alpha diversity indices, including the ACE, Chao1, Simpson and Shannon indices, reflect the richness and diversity of microorganisms in materials and fermented feed. The Chao1 and ACE indices were used to measure bacterial abundance in this study, while the Shannon and Simpson indices were used to measure species diversity. As shown in Fig. 1, FMB and WMB fermented feed had higher bacterial species abundance and diversity. This may be because the moisture content of the two materials was 61–79%, which is suitable for the growth of aerobic microorganisms in an aerobic environment. After ensiling, some Gram-negative bacteria with thin cell walls died as fermentation progressed in the anaerobic and acidic environment. Finally, only Gram-positive bacteria with thick cell walls, such as LAB species, can grow in ensiling process and promote lactic acid fermentation [44]. This was the main reason why LAB species replaced the harmful microorganisms in the material and became the dominant community, which led to a marked decrease in microbial diversity after ensiling.

Venn diagrams clearly show the distribution of common and unique microorganisms in mulberry materials and fermented feed. Before ensiling, the relatively high moisture content in the FMB material was more suitable for the growth of aerobic microorganisms, resulting in a higher number of OTUs compared with wilted material (Fig. 2). After ensiling, LAB produced lactic acid to reduce the pH and inhibit the growth of other microorganisms, thereby reducing microbial diversity. The LAB inoculation and cellulase enzymes both promoted lactic acid fermentation, leading to a decrease in microbial diversity.

### Relative abundance of bacteria at the genus and species levels in the material and fermented feed

Anaerobic fermentation is a highly complex process involving microbial activity and biochemical changes. The quality of fermented feed depends on the succession of the microbial community and changes of microbial metabolites during the ensiling process. The predominant microbial community in the mulberry material used in this study was *Achromobacter* (Fig. 3), a non-fermenting Gram-negative bacterium resistant to many antibiotics such as most cephalosporins, aztreonam and aminoglycosides [45]. Another dominant bacterium is *A. muciniphila*, which is a Gram-negative bacterium commonly present in the human gut and accounting for 3–5% of the human microbial community. It grows in the intestinal mucus layer and feeds on mucins secreted by the host, and colonizes and protects the gut from pathogens through competitive exclusion [46]. Although *Akkermansia* uses mucin as an energy source, numerous observations have confirmed that it regulates the thickness of the intestinal mucus layer and integrity of the intestinal barrier [47]. *Alistipes*, another dominant bacterium, is associated with fibre degradation in the rumen and utilizes degraded soluble sugars as fermentation substrates [48]. *Caproiciproducens* spp. produce carbon dioxide, ethanol, acetic acid and butyric acid under anaerobic conditions [49]. These dominant bacteria are not common during the anaerobic fermentation process and their roles in fermented feed preparation remain unclear. However, *Achromobacter* exhibits pathogenicity and drug resistance [45], which may have adverse effects on fermented feed and livestock. *A. muciniphila* may regulate the intestinal environment in livestock [50], while *Alistipes* can decompose fibres in the rumen [48]. *Caproiciproducens* can generate volatile fatty acids, which play a role in the preparation of fermented feed and maintenance of livestock health [51]. In this study, *L. plantarum* was the dominant species in all fermented feed, especially in wilting fermented feed, in which it had the highest relative abundance. Appropriate adjustment of the moisture of the mulberry material by wilting likely promoted vigorous proliferation of LAB. The addition of LAB inoculant markedly improved the quality of fermented feed. It is possible that the *L. plantarum* used in this study had strong acid resistance. During ensiling, this bacterium can rapidly respond to the stress imposed by the anaerobic and acidic fermentation environment, and promote lactic acid fermentation [8, 52].

In this study, *P. agglomerans* were dominant in FMB material, and* P. agglomerans* and *M. adhaesivum* were dominant in WMB materials. *P. agglomerans* is a Gram-negative bacillus that occurs widely in nature. It is usually found in plants, as epiphytes or endosymbionts, and can be isolated from water, human wounds, blood and urine. It is a pathogen of cultivated plants and one of the causative bacteria of bacterial wilt [53]. Therefore, this bacterium should be considered harmful during ensiling. *Methylobacterium* is an aerobic, mesophilic, Gram-negative, facultative methylotrophic bacterium isolated from drinking water [54]; it is mainly distributed on soil and plant surfaces. *Methylobacterium* can use methanol and methylamine, and compounds C2–C4, to promote plant growth, and forms root nodule nitrogen-fixing symbionts with legumes such as rhizobium [55]. It rarely occurs in forage materials and its role in anaerobic fermentation is not clear. However, after ensiling, LAB proliferate rapidly, quickly replacing the above two bacteria and other harmful microorganisms as the dominant bacteria. *L. plantarum* is an anaerobic or facultative anaerobic Gram-positive bacteria that belongs to the homofermentative LAB; its main metabolite is lactic acid [10]. In the anaerobic silo environment, LAB can decompose sugars to produce a large amount of lactic acid, which reduces the pH value of the fermented feed. The acidic environment formed by lactic acid fermentation kills spoilage bacteria and clostridia during ensiling [14]. Due to the accumulation of lactic acid and enhanced acidity, anaerobic fermentation inhibits not only other harmful microorganisms, but also the activity of LAB, thereby enabling fermentation to reach a stable state [56].

In this study, wilting effectively adjusted the moisture content of mulberry, thereby promoting lactic acid fermentation. The addition of LAB or cellulase alone improved the quality of mulberry fermented feed, and the two additives in combination had a synergistic effect. The cellulase used in this study is produced by *A. cellulolyticus* and includes endoglucanases, cellobiohydrolases and beta-glucosidases. These cellulases decompose plant cell walls such that cellular contents are released, thereby reducing the fibre content of forage material. In addition, cellulase can degrade the carbohydrates in material into monosaccharides or disaccharides [57]. In this study, the epiphytic LAB counts and WSC content in mulberry were low, which led to low-quality natural silage fermentation. The cellulolytic enzyme used in this study can provide sufficient fermentation substrates for LAB, and inoculated *L. plantarum* can utilize these fermentation substrates to produce lactic acid through the glycolytic EMP pathway. The synergistic effect of cellulase and LAB was responsible for the improvement in quality of mulberry fermented feed. Cellulolytic enzymes provide sufficient fermentation substrates for silage fermentation, which microorganisms, including clostridia associated with silage fermentation, can use to grow when conditions are suitable. However, in an anaerobic silage environment, LAB are usually the dominant bacteria and are the main utilizers of these substrates. LAB use these sugars to produce lactic acid and lower the pH for long-term storage [58].

*Streptococcus mitis* and *C. tyrobutyricum* were relatively abundant in the control fermented feed of FMB and WMB. *S. mitis* is a common species in the oropharynx, skin, digestive system and female reproductive system, and is not a pathogenic bacteria [59]. Like *Enterococcus* species, this bacterium is commonly found in fermented feed, possibly due to human contamination during anaerobic fermentation and storage. In this study, the pH of the mulberry control fermented feed was > 5, which was suitable for the growth of *C. tyrobutyricum*. They competed with LAB for nutrients, resulting in a decrease in the *L. plantarum* count. In addition, the growth of this bacterium can cause fermented feed DM loss, protein degradation, and an accumulation of ammonia, amines, and amides, thereby reducing the quality of fermented feed [60]. Clostridia are the Gram-positive and anaerobic bacteria found mainly in soil, human and animal intestines and spoilage. They have endospores and can grow in anaerobic and high-temperature environments [61]. Some of these clostridia are pathogenic, such as *Clostridium tetani* and *Clostridium botulinum*, which produce exotoxins and invasive enzymes [62]. Clostridia can be mixed into the feed with the soil during silage preparation and can multiply in large numbers in environments where the silage pH is not less than 4.2, reducing the quality of the fermented silage and at the same time risking the health of livestock [14]. Therefore, the effective use of microbial agents to inhibit *Clostridium* during silage fermentation is critical to the success or failure of high-quality silage. In this study, LAB and cellulolytic enzymes acted synergistically to not only promote lactic acid fermentation, but also inhibit the growth of clostridia. This is important for improving the fermentation quality of silage and ensuring feed and livestock safety.

### KEGG pathways in the material and fermented feed

Metabolic gene clusters participate in multiple secondary metabolic pathways, and are also involved in nutrient acquisition, toxin degradation, antimicrobial resistance, and vitamin biosynthesis [63]. During anaerobic fermentation, these metabolic gene clusters can affect the metabolism and metabolites of fermented feed microorganisms, thereby influencing the quality of silage fermentation. In the present study, GOM was the main metabolic category of microorganisms in all mulberry materials and fermented feed (Fig. 4). The GOM metabolic category involves secondary metabolite biosynthesis, microbial metabolism in different environments, carbon metabolism, 2-oxocarboxylic acid metabolism, fatty acid metabolism, amino acid biosynthesis and degradation of aromatic compounds. During ensiling, the GOM metabolic category became more dominant due to the high relative abundance of LAB and enhancement of their secondary metabolic pathways. In this study, GOM, amino acid metabolism and carbohydrates emerged as the dominant metabolic categories in silage. According to KEGG pathway database, GOM includes metabolic pathways such as biosynthetic metabolic pathways of secondary metabolites, microbial metabolism in diverse environments, carbon metabolism, 2-oxocarboxylic acid metabolism, fatty acid metabolism, amino acid biosynthesis and degradation of aromatic compounds [64]. However, the effects of silage fermentation and the synergistic effects of enzyme–LAB on these metabolic pathways are not well clear and need to be further explored in the future. Essential and non-essential amino acids are produced by protein hydrolysis. The nutritional value of proteins for ruminants depends on the type, quantity, and proportion of essential amino acids [65]. We observed a diverse range of epiphytic microorganisms in mulberry material; some microorganisms were able to decompose protein, resulting in the dominance of amino acid metabolic pathways in material relative to fermented feed. The synergistic enzyme–bacteria effect effectively promoted lactic acid fermentation, but inhibited the growth of harmful bacteria and protein hydrolysis, thereby decreasing the dominance of amino acid metabolic pathways. During the ensiling process, the proliferation of LAB increased the dominance of the WSC metabolic pathway in the material, and generated lactic acid, reduced the fermented feed pH, and improved the quality of the fermented feed. The dominant carbohydrate metabolic pathways in fermented feed were as follows: “additive combined fermented feed” > “additive alone fermented feed” > control fermented feed > “material”.

### Microbial community, terminal fermentation products, and the co-occurrence network

Many kinds of microorganisms are involved in silage fermentation. They produce different metabolites through different metabolic pathways, and directly affect fermentation quality [15]. For example, *L. plantarum* is a homofermentative LAB that uses glucose to produce lactic acid, while *Leuconostoc* spp. are a type of heterofermentative LAB that use glucose to produce lactic acid, carbon dioxide and ethanol. *Enterococcus* spp. avoid the tricarboxylic acid metabolic pathway that ferments lactic acid to produce acetate [8, 66]. These metabolites also affect the microbial community structure during ensiling. The organic acids produced by anaerobic fermentation microorganisms reduce the pH and inhibit the growth of aerobic bacteria and moulds, which not only improve the fermentation quality, but also play a role in preventing the aerobic deterioration of fermented feed. In this study, *L. plantarum* and *L. kullabergensis* may have utilized WSC to produce lactic acid and lower the pH; they were positively correlated with WSC and negatively correlated with pH (Fig. 5). *Clostridium* can resist the anaerobic and acidic environmental conditions of anaerobic fermentation, compete with LAB for nutrients, and inhibit lactic acid fermentation. *Muribaculaceae* spp. have been observed in the mammalian gut [67]. This is the first study to report these bacteria in mulberry fermented feed, and its role in anaerobic fermentation is still unclear. Woody plants are rich in macro-minerals and usually have a high LBC, which negatively affects silage fermentation [3]. As shown in Table 1, the mulberry used in this study was rich in cations such as K^+^, Ca^2+^ and Mg^2+^, and had a high LBC value. These cations neutralize the acidic environment formed by lactic acid fermentation during ensiling, which can easily lead to the proliferation of harmful bacteria [14]. This could explain why LBC was negatively correlated with *L. plantarum* and *Lactobacillus kullabergensis*, and positively correlated with harmful bacteria such as *Clostridium saccharolytica* and *Pseudomonas* spp.

*P. agglomerans*, *M. testaceum* and *A. muciniphila* are Gram-negative bacteria that negatively affect anaerobic fermentation [68]. *Enterococcus* is a Gram-positive lactic acid-producing cocci that is widely distributed in the natural environment and occurs in the digestive tract of humans and animals [69]. Some enterococci can produce bacteriocin and other antibacterial substances in the anaerobic fermentation environment, which inhibits the growth of pathogenic bacteria such as *Escherichia coli* and *Salmonella* spp., and improves fermentation quality [26]. In the early stage of anaerobic fermentation, *Enterococcus* species multiply vigorously, inhibit harmful bacteria by producing lactic acid and bacteriocins, and create an environment promoting the subsequent growth of *Lactobacillus* species, and *L. plantarum* has strong acid resistance and proliferates in the anaerobic fermentation environment, thereby inhibiting the growth of other harmful bacteria [70]. In this study, *L. plantarum* had a positive correlation with *Enterococcus* species and negative correlation with the three harmful bacteria (Fig. 6).

## Conclusions

This study used the SMRT sequencing technology to explore the microbial community, functional genes, co-occurrence network of mulberry, and the synergistic effect of cellulase and LAB on the silage fermentation. Cellulase and LAB exert synergistic effects during woody anaerobic fermentation, accelerating the dynamic succession of the dominant bacteria from Gram-negative to Gram-positive, and forming a co-occurrence microbial network with LAB at the core. Both the wilting and microbial inoculant preparation methods increased the dominance of the GOM and carbohydrate metabolism categories, but reduced that of amino acid metabolism category; they also improved the fermentation quality of mulberry silage.

## Data Availability

All data generated or analysed during this study are included in this article.

## Abbreviations

LAB: Lactic acid bacteria
WSC: Water-soluble carbohydrate
SMRT: Single-molecule real-time
AC: *Acremonium cellulase*
CH: *Lactiplantibacillus* *plantarum*
FM: Fresh matter
cfu: Colony-forming unit
DM: Dry matter
CP: Crude protein
EE: Ether extract
OM: Organic matter
ADF: Acid detergent fibre
NDF: Neutral detergent fibre
ADL: Acid detergent lignin
NH_3_-N: Ammonia nitrogen
KEGG: Kyoto Encyclopedia of Genes and Genomes
LBC: Lactic acid buffering capacity
FMB: Fresh mulberry
WMB: Wilted mulberry
OTU: Operational taxonomic unit
CCS: Circular consensus sequencing
